# Self-assessment in laparoscopic surgical skills training: Is it reliable?

**DOI:** 10.1007/s00464-016-5246-6

**Published:** 2016-09-21

**Authors:** Sandeep Ganni, Magdalena K. Chmarra, Richard H. M. Goossens, Jack J. Jakimowicz

**Affiliations:** 1 Delft University of Technology, Industrial Design Engineering, Medisign, Delft, The Netherlands; 2 Department of Surgery, GSL Medical College, Rajahmundry, India; 3 Research and Education, Catharina Hospital, Michelangelolaan 2, 5653 EJ Eindhoven, The Netherlands

**Keywords:** Self-assessment, Expert-assessment, Laparoscopic cholecystectomy, Training, Evaluation, Laparoscopic skills

## Abstract

**Background:**

The concept of self-assessment has been widely acclaimed for its role in the professional development cycle and self-regulation. In the field of medical education, self-assessment has been most used to evaluate the cognitive knowledge of students. The complexity of training and evaluation in laparoscopic surgery has previously acted as a barrier in determining the benefits self-assessment has to offer in comparison with other fields of medical education.

**Methods:**

Thirty-five surgical residents who attended the 2-day Laparoscopic Surgical Skills Grade 1 Level 1 curriculum were invited to participate from The Netherlands, India and Romania. The competency assessment tool (CAT) for laparoscopic cholecystectomy was used for self- and expert-assessment and the resulting distributions assessed.

**Results:**

A comparison between the expert- and self-assessed aggregates of scores from the CAT agreed with previous studies. Uniquely to this study, the aggregates of individual sub-categories—‘use of instruments’; ‘tissue handling’; and errors ‘within the component tasks’ and the ‘end product’ from both self- and expert-assessments—were investigated. There was strong positive correlation (*r*_s_ > 0.5; *p* < 0.001) between the expert- and self-assessment in all categories with only the ‘tissue handling’ having a weaker correlation (*r*_s_ = 0.3; *p* = 0.04). The distribution of the mean of the differences between self-assessment and expert-assessment suggested no significant difference between the scores of experts and the residents in all categories except the ‘end product’ evaluation where the difference was significant (*W* = 119, *p* = 0.03).

**Conclusion:**

Self-assessment using the CAT form gives results that are consistently not different from expert-assessment when assessing one’s proficiency in surgical skills. Areas where there was less agreement could be explained by variations in the level of training and understanding of the assessment criteria.

The concept of self-assessment has been widely acclaimed for its role in professional development cycle and self-regulation [1, 2]. The term self-assessment itself, however, is loosely defined and is thus the subject of criticism regarding its effectiveness in practice [3]. There has been considerable debate as to the efficacy of self-assessment but most criticism of self-assessment concerns the methodologies used, rather than the pedagogy itself [4–6]. Several educational psychology studies assert that self-assessment should be integrated from within the training phase to inculcate it as a lifelong professional habit [7–9]. In professional practice, however, the reality is that self-assessment is most commonly used as an evaluative tool for final performance [10].

In the field of medical education, self-assessment is mostly used to evaluate the cognitive knowledge of students [11, 12]. In surgical training, where acquisition of complex surgical skills such as cognitive, psychomotor and decision-making skills is required, self-assessment has not gained enough attention. In laparoscopic surgery, assessment of surgical skills is done either by surgical experts or by means of virtual reality (VR) simulators [13, 14]. Though VR simulators offer a certain degree of self-assessment, it is limited to psychomotor skills assessment against pre-defined benchmarks [15].

In addition to the complexity of assessment of skills in laparoscopic surgery, the costs—in terms of actual hours and time spent away from the operating theatre—of training and evaluating surgical residents by expert are very high [16]. An effective self-assessment tool could help in reflection on performance and assessment of trainees in the course of training and thus sequentially reducing the workload of expert surgeons.

The aim of this study was to assess the validity of using self-assessment within the Laparoscopic Surgical Skills curriculum (an initiative of the European Association of Endoscopic Surgery) [17]. The competency assessment tool (CAT) for laparoscopic cholecystectomy (LC) was used for self-assessment and expert-assessment in this study, and the results were compared.

## Materials and methods

### Participants

Thirty-five surgical residents who attended the 2-day Laparoscopic surgical skills Grade 1 Level 1 curriculum were invited to participate (Table 1). Their expertise level ranged from PGY-2 to PGY-3. All of the surgical residents had prior experience using both box trainers and VR simulators.

**Table 1:** Demographic data of participants

	Eindhoven, The Netherlands	Cluj-Napoca, Romania	Rajahmundry, India	Total
Male	4	3	11	18
Female	5	2	10	17
Total	9	5	21	35

All participants voluntarily enrolled in the study and signed an informed consent prior to the start of the curriculum. They also had to fill in a demographic questionnaire with data pertaining to experience in laparoscopic surgery and time spent preparing for the curriculum.

Six expert surgeons from the respective locations conducting the curriculum were invited to participate as expert assessors. Their experience in laparoscopic surgery ranged from 5 to 25 years, each with more than 200 laparoscopic procedures performed as a main surgeon. They also all had experience using the CAT form as a form of evaluation previously.

### Task

The participants had to fill out a multiple choice questionnaire on the basics of laparoscopic surgery to be admitted into the curriculum. During the curriculum, they participated in interactive discussions on the basics of laparoscopic surgery and LC, training on VR simulators and box trainers.

Each participant performed an LC procedure on a pig liver placed in a box trainer. The box trainer with ports that mimicked incision points was placed on a height adjustable table with monitors and equipment in place. Each participant was assisted by a fellow participant, who held the camera and, when needed, the instruments: playing the role of an assistant. The expert surgeons instructed the participants on the procedural tasks prior to the procedure and intervened whenever they deemed instruction was necessary. However, the assessors were asked not to express their opinions on the performance whilst the participants performed the procedure. After completing the procedure, both the participants and expert surgeons had to fill in the CAT form independently of one another.

### Assessment

The CAT form was used in the study for self-assessment and expert-assessment. The CAT is an operation-specific assessment tool that was adapted for the LC procedure for use within the curriculum [18]. The evaluation criteria are spread across three procedural tasks: exposure of cystic artery and cystic duct, cystic pedicle dissection and resection of gallbladder from the liver. Within these tasks, the performance was rated on a five-point task-specific scale based on the usage of instruments, handling of tissue with the non-dominant hand (NDH), errors within each task and the end product of each task.

### Statistical analysis

Analysis was done comparing the expert- and self-assessment scores based on the above-mentioned criteria within the tasks. Scores for each category were summed to form aggregate scores for each, related, category. The scores for all the criteria were also calculated in order to compare our results with other studies. Obtained data were analysed using GraphPad Prism (Version 7.00). Spearman’s rank correlation was used to assess the correlation between the expert- and self-assessment results. The Wilcoxon matched-pairs signed-rank test was used to assess whether the population mean ranks differ. A *p* value of <0.05 was considered statistically significant.

## Results

### Correlation is seen between expert- and self-assessment

**Fig. 1:**
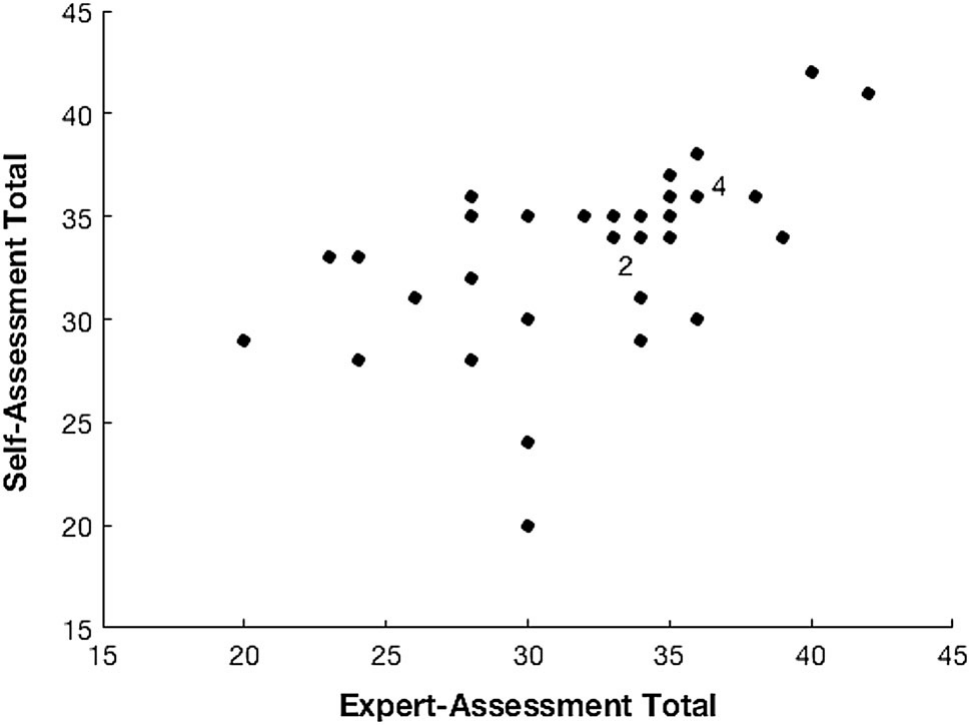
Self-assessment (SA) versus expert-assessment (EA) score for aggregated responses to all questions. *Numbers* to the right of data points show the number of coincident data points at the same coordinates, i.e., the number of people with the same combination of SA and EA scores

**Fig. 2:**
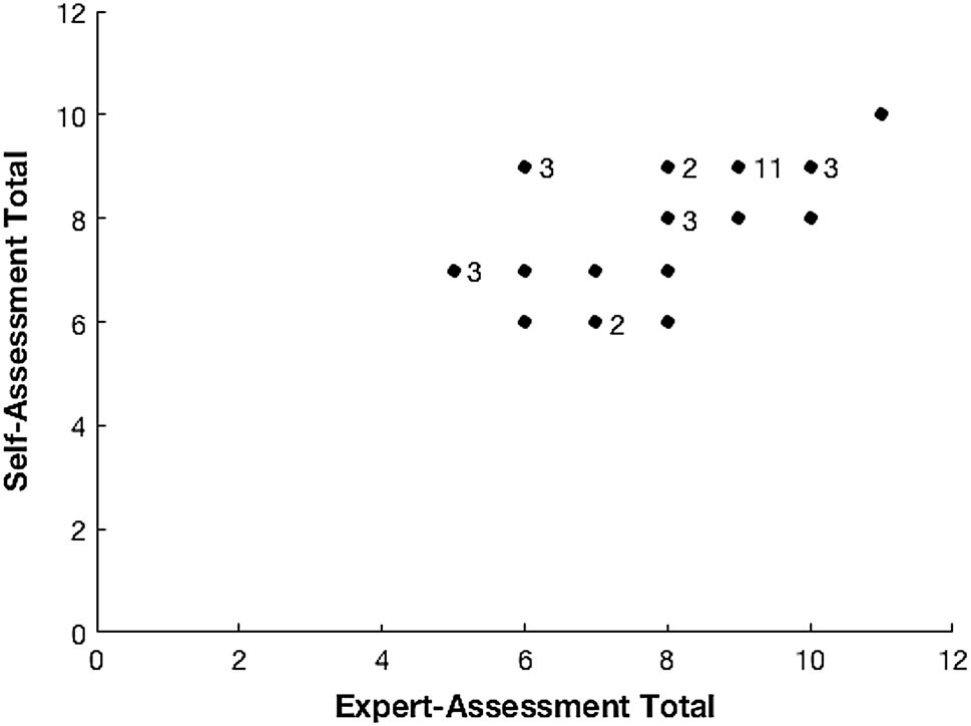
Self-assessment (SA) versus expert-assessment (EA) score for aggregated responses to ‘usage of instruments’ questions. *Numbers* to the right of data points show the number of coincident data points at the same coordinates, i.e., the number of people with the same combination of SA and EA scores

Figures 1 and 2 show exemplar scatter plots for the aggregate scores of the all criteria and tissue-handling data, respectively. There is statistically significant positive correlation between self-assessed answers and expert’s opinions. All groupings show a Spearman’s rank of greater than 0.5, corresponding to a strong positive correlation with the exception of the tissue handling and usage of NDH grouping which shows a weaker positive correlation of 0.3042.

### Similar distribution of responses between expert- and self-assessment

**Fig. 3:**
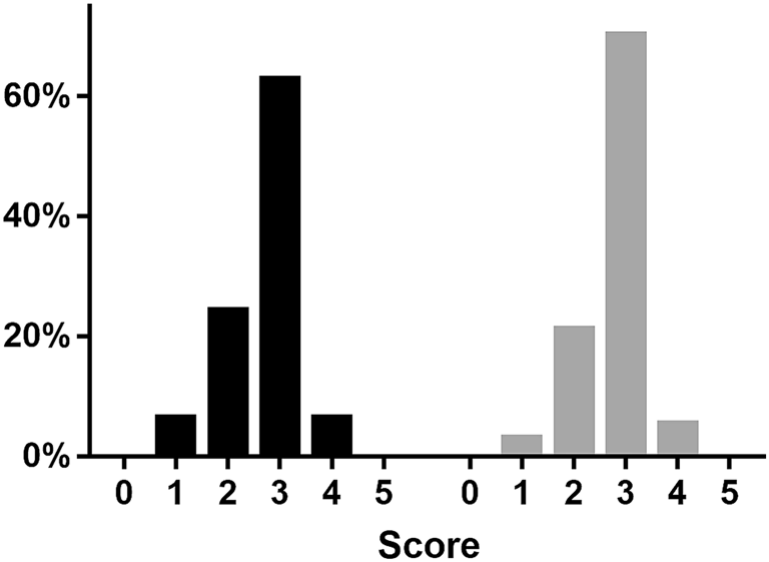
Percentage histogram showing the (qualitative similarity of the) overall distribution of responses from expert-assessment (*black*) and self-assessment (*grey*)

**Fig. 4:**
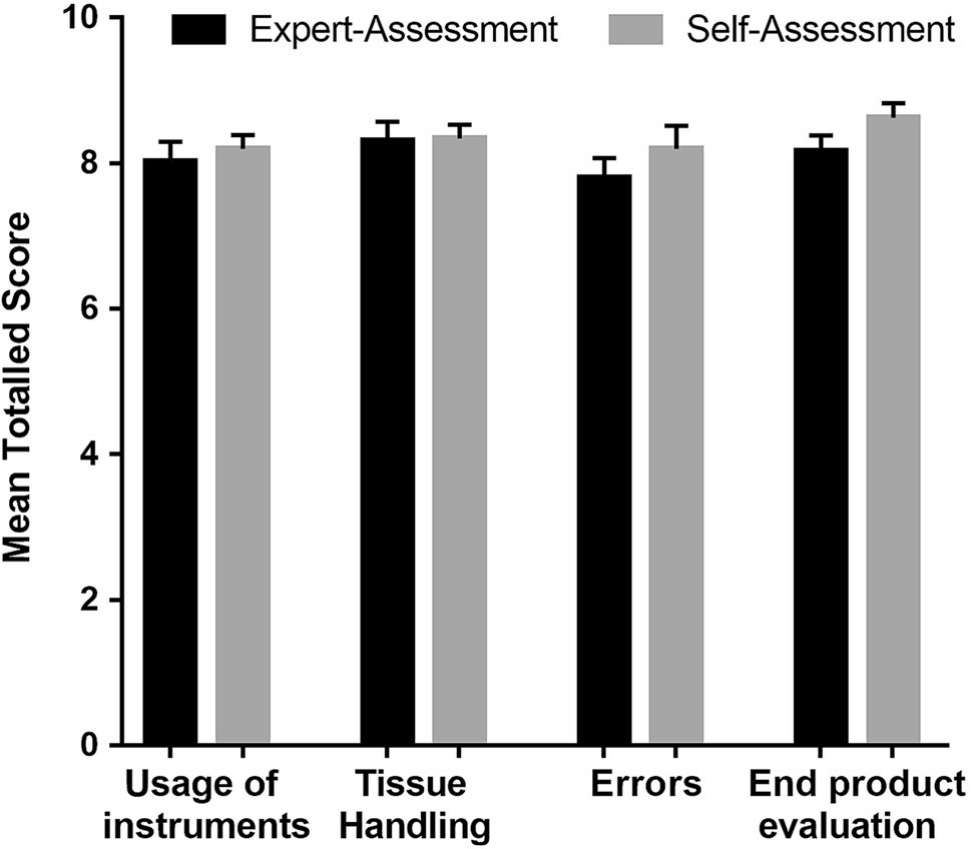
Mean ± SEM for the expert-assessment (*black*) and the self-assessment (*grey*) total score for the four question groups described on the *x*-axis

The statistics calculated to compare their distribution are shown in Table 2. Figure 3 shows the distribution of the responses of both experts and participants. Figure 4 demonstrates how similar the means (±SEM) of the grouped, aggregated data are. With the exception of the ‘end product evaluation’ criterion, all the groupings result in a *p* value greater than the 0.05 threshold for rejecting the null hypothesis. The ‘end product evaluation’ criterion has a Wilcoxon *p* value of 0.0339 which suggests that in the case of the ‘end-product evaluation’ criterion a difference in the distribution of the mean difference was seen. Furthermore, there was no significant difference between the mean of the differences in scores for men (1.17, SD = 3.32; SEM 0.76) and women (0.94, SD = 5.60; SEM 1.37) whose demographic distribution can be seen in Table 1.

**Table 2:** Statistics comparing overall and grouped self-assessment with expert-assessment

Criteria	Mean of expert-assessment (SD; SEM)	Mean of self-assessment (SD; SEM)	Spearman’s rank correlation (*p* value)	Sum of signed ranks (*W*) (*p* value)
All criteria	32.31 (5.05; 0.85)	33.37 (4.31; 0.73)	0.6431 (<0.0001*)	116 (0.1667)
Usage of instruments	8.03 (1.60; 0.27)	8.20 (1.13; 0.19)	0.6208 (<0.0001*)	38 (0.4760)
Tissue handling and usage of NDH	8.31 (1.53; 0.26)	8.34 (1.1; 0.19)	0.3042 (0.0378*)	35 (0.9753)
Errors	7.80 (1.62; 0.27)	8.20 (1.86; 0.31)	0.5376 (0.0004*)	87 (0.1888)
End-product evaluation	8.17 (1.27; 0.21)	8.62 (1.17; 0.20)	0.5180 (0.0007*)	119 (0.0339*)

* Statistically significant result

## Discussion

In surgical education, due to the complex structure of training and evaluation, several studies have explored the reliability of self-assessment using various methodologies [5, 11, 19]. In the past decade, VR simulators have gained significance in surgical skills training and assessment; and a number of studies prove that they provide feedback that is quite essential for the participants to self-assess their performance [20, 21]. For surgical specialties self-assessment to be more accurate, Mandel et al. [22] suggest that the use of task specific and global check lists should be incorporated. Moreover, as Kostons et al. [7] mentioned in their review on self-assessment, when concurrent monitoring is hampered, that is likely over a period of time, learners have poor recollection of their performance which in turn may hamper their self-assessment after the task.

The objective of this study was to encompass the findings of these prominent studies in surgical training and incorporate them into the study design. Whilst these studies have established the importance of self-assessment as a methodology and its role in education and training, this is the first which has focussed on evaluating performance in individual components of the task. Therefore, the surgical residents were trained on VR simulators, self-assessment was done immediately after the procedure using the CAT form, and they participated in a curriculum that detailed the procedural tasks of the LC.

Evaluating the responses to all components taken together agreed with previous studies: there is a strong correlation between the aggregated responses to the evaluation given by the participants and experts. Evaluating individual procedural tasks independently allowed for individual insights on the strengths and weaknesses in performance and evaluation. The fact that the results indicated a strong correlation between expert- and self-assessment in terms of the ‘use of instruments’ category could be attributed to the training on VR simulators and box trainers prior to the procedure. A strong correlation found in the evaluation of ‘errors’ category might indicate a clear layout of errors in the CAT form. Evaluating the distribution of differences leads to no significant differences between the means of the distribution except in the case of the end-product evaluation.

The weaker correlation in terms of tissue handling and usage of NDH could probably be explained by difficulties in observing the NDH, as most surgeons are inclined to look at the actions performed with their dominant hand. The significant difference in the difference of means in the ‘end point evaluation’ may be attributed to lack of adequate focus on these aspects during the curriculum. Overall, however, the distribution of self-assessment scores is similar and well correlated with expert-assessment. This suggests that self-assessment is a reliable tool to assess one’s own performance.

The limitation of our study was the lack of consistent instruction on the usage of the CAT tool to the participants prior to self-assessment. A few studies suggest that surgical residents are better able to self-assess their performance after they have watched benchmark videos; moreover, courses concentrated on the procedural skills of the task have been shown to significantly improve the outcomes of the self-assessment of surgical residents [23, 24].

We intend to explore further how self-assessment is integrated into surgical curricula and, in particular, to investigate whether providing videos and/or images as reference for those conducting self-assessment could improve the efficacy of self-assessment in the areas we found to be less matched with expert-assessment. This in turn could prove beneficial in providing more accurate formative and summative self-assessment in laparoscopic surgical skills.

## Conclusion

Provided that there is proper understanding and training of the evaluation criteria beforehand, self-assessment using the CAT form gives results that are consistently not different from expert-assessment when assessing one’s proficiency in surgical skills. Areas where there was less agreement could be explained by variations in training.
